# Multi-Omic Analysis Reveals the Potential Anti-Disease Mechanism of Disease-Resistant Grass Carp

**DOI:** 10.3390/ijms26083619

**Published:** 2025-04-11

**Authors:** Chongqing Wang, Zeyang Li, Xu Huang, Xidan Xu, Xiaowei Xu, Kun Zhang, Yue Zhou, Jinhai Bai, Zhengkun Liu, Yuchen Jiang, Yan Tang, Xinyi Deng, Siyang Li, Enkui Hu, Wanjing Peng, Ling Xiong, Qian Xiao, Yuhan Yang, Qinbo Qin, Shaojun Liu

**Affiliations:** 1 Engineering Research Center of Polyploid Fish Reproduction and Breeding of the State Education, Ministry, College of Life Sciences, Hunan Normal University, Changsha 410081, China; wcq@hunnu.edu.cn (C.W.); 15310949170@163.com (Z.L.); xiuhuang1993@163.com (X.H.); 15377324945@163.com (X.X.); xgx1921192165@163.com (X.X.); 15034676496@163.com (K.Z.); taixiuvv@outlook.com (Y.Z.); b13807253562@outlook.com (J.B.); l1530697267@outlook.com (Z.L.); yc990213@163.com (Y.J.); 18311508595@163.com (Y.T.); q2819733767@163.com (X.D.); lsy430181@126.com (S.L.); 17680540702@163.com (E.H.); 19173996279@163.com (W.P.); xiongling@hunnu.edu.cn (L.X.); xiaoqian_0225@163.com (Q.X.); ayyhmf@126.com (Y.Y.); lsj@hunnu.edu.cn (S.L.); 2 Nansha-South China Agricultural University Fishery Research Institute, Guangzhou 511457, China

**Keywords:** gut–liver axis, disease-resistant grass carp, intestinal microflora, metabolites, transcriptome

## Abstract

The gut–liver axis is essential in animal disease and health. However, the role of the gut–liver axis in the anti-disease mechanism of disease-resistant grass carp (DRGC) derived from the backcross of female gynogenetic grass carp (GGC) and male grass carp (GC) remains unclear. This study analyzed the changes in gut histopathology, fecal intestinal microflora and metabolites, and liver transcriptome between GC and DRGC. Histological analysis revealed significant differences in the gut between DRGC and GC. In addition, microbial community analyses indicated that hybridization induced gut microbiome variation by significantly increasing the proportion of Firmicutes and Bacteroidota in DRGC. Metabolomic data revealed that the hybridization-induced metabolic change was probably characterized by being related to taurocholate and sphinganine in DRGC. Transcriptome analysis suggested that the enhanced disease resistance of DRGC was primarily attributed to immune-related genes (*SHMT2*, *GOT1*, *ACACA*, *DLAT*, *GPIA*, *TALDO1*, *G6PD*, and *FASN*). Spearman’s correlation analysis revealed a significant association between the gut microbiota, immune-related genes, and metabolites. Collectively, the gut–liver axis, through the interconnected microbiome–metabolite–gene pathway, may play a crucial role in the mechanism of greater disease resistance in DRGC, offering valuable insights for advancing the grass carp cultivation industry.

## 1. Introduction

The intestinal microorganism is believed to function as an additional organ [1,2], and the microbiome significantly contributes to the maintenance of biological processes in the host [3]. Growing research on host–microbe interactions has demonstrated that gut microbiota plays a vital role in host health and immune function [4,5]. Numerous factors, such as habitat environment, season, host genetics, developmental stage, nutrition level, and diet composition, have been reported to influence the origin and composition of fish gut microbiota, with the habitat environment potentially being the primary determinant [6].

Furthermore, alterations in gut bacteria, driven by the host’s genetics, could modify fish immunity and their ability to resist diseases [7,8]. In general, the gut microbiome plays a central role in regulating host immunity through the connection between the intestines and the liver [9]. The liver receives blood from the intestine and is profoundly impacted by the gut microbiota and its metabolites [10]. Hence, a close association is observed between the liver and the intestine, describing the gut–liver axis [11]. Growing evidence in animal research indicates that the gut–liver axis affects the host’s health and disease [12].

Fish represents a high-quality animal protein source for human consumption [13]. In the aquaculture industry, grass carp (*Ctenopharyngodon Idella*, GC) are among the most extensively cultivated freshwater fish worldwide, contributing greatly to aquaculture production [14]. In China, grass carp rank first in terms of output and economic value in freshwater aquaculture [15]. However, GC are susceptible to microbial invasion due to high industrial density and intensive farming practices, increasing vulnerability to disease outbreaks [16]. The primary GC diseases include viral hemorrhagic disease and recurring bacterial infections from seasonal bacteria, leading to major economic losses in aquaculture [17]. Recent studies have investigated the effects of enhancing feed ingredients to boost immunity in GC, such as incorporating antimicrobial peptides, gut probiotics, dietary choline, curcumin meal, and developing vaccines [18,19,20,21,22]. Notably, genetic breeding methods were employed to create new strains of grass carp with greater disease resistance and faster growth, including gynogenetic grass carp [23], genetically selected grass carp “Husu No.2” [24], and hybrid grass carp [25]. In the last few years, our team has produced disease-resistant grass carp (DRGC) by backcrossing female gynogenetic grass carp (GGC) with male GC, resulting in improved survival, growth, and disease resistance [26]. However, the role of the gut–liver axis in disease resistance in DRGC remains unexplored.

This research investigated the possible anti-disease mechanisms in disease-resistant grass carp by analyzing the gut histology, microbiota, metabolome, and liver transcriptome. The intestine of both DRGC and GC was subjected to histological analysis, and the microbiota and metabolome were determined. Transcriptome analysis was conducted on liver tissues from both groups of grass carp. This study elucidates the potential anti-disease mechanism in disease-resistant grass carp, enhancing aquaculture productivity and economic efficiency while supporting environmental protection and food safety, and providing essential data for grass carp immunology research.

## 2. Results

### 2.1. Intestinal Histology

**Figure 1:**
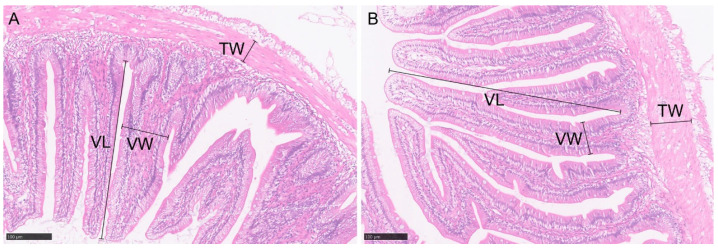
Intestinal histology in grass carp (GC) (**A**) and disease-resistant grass carp (DRGC) (**B**). VL: villus length; TW: thickness of gut wall; VW: villus width. Scale bars: 100 μm.

Figure 1 presents the H&E staining of the intestinal tissues of DRGC and GC. The DRGC group exhibited significantly increased villus length and gut wall thickness compared to the GC group (*p* < 0.05), as shown in Table 1. The villus width was significantly greater in the GC group than in the DRGC group (*p* < 0.05).

### 2.2. 16S rRNA Sequencing Analysis and Taxonomic Annotation

**Figure 2:**
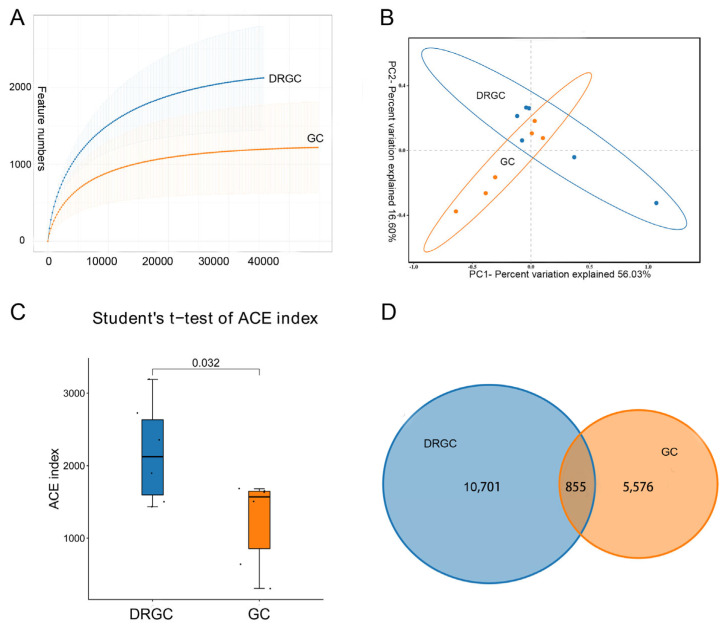
Rarefaction curves of grass carp (GC) and disease−resistant grass carp (DRGC) intestinal microbial samples (**A**); principal coordinate analysis (PCoA) of samples from two groups (**B**); alpha diversity based on the ACE index of the operational taxonomic unit (OTU) level (**C**), n = 6; and Venn diagram of OTU distribution (**D**).

A comprehensive analysis of all intestinal samples yielded 855,682 effective tags (Table S1). The sequence sparse curve revealed that the sample size adequately represents community richness, and further data analysis was performed (Figure 2A). The principal coordinate analysis (PCoA) indicated a greater degree between GC and DRGC (Figure 2B). A difference in the α diversity index of intestinal microbiota was found between the DRGC and GC groups (Figure 2C) (*p* < 0.05). In the DRGC group, 11,556 operational taxonomic units (OTUs) were identified, including 10,701 unique OTUs, while the GC group had 6431 OTUs, among which 5576 were unique (Figure 2D).

### 2.3. Intestinal Microbial Composition

**Figure 3:**
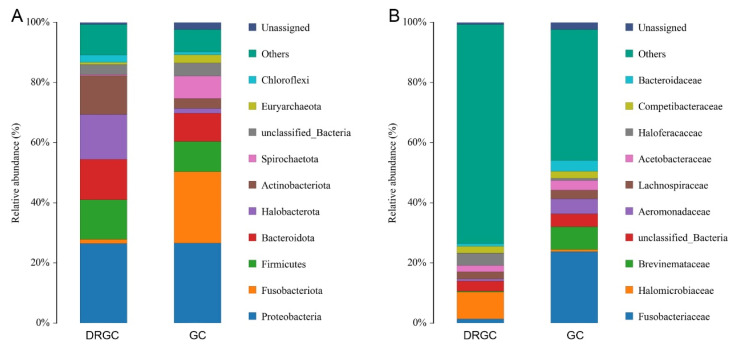
Relative abundances of dominant bacterial phyla (**A**) and families (**B**) in the intestines of disease-resistant grass carp (DRGC) and grass carp (GC) groups.

**Figure 4:**
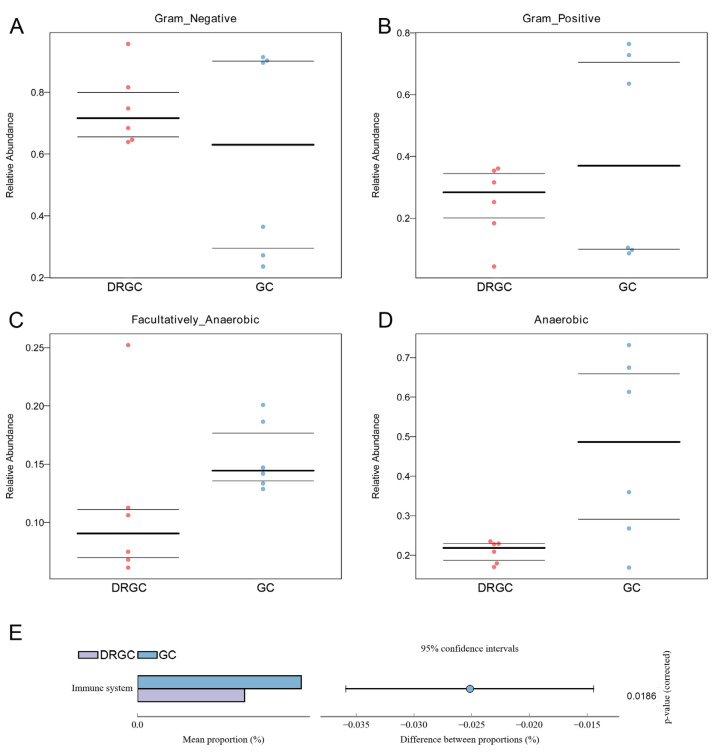
Organism-level microbiome phenotypes were predicted by Bugbase (**A**–**D**). Heatmap of functional prediction by PICRUSt (**E**). DRGC: disease-resistant grass carp; GC: grass carp.

Among all intestinal samples, Firmicutes and Proteobacteria were the predominant taxa (Figure 3A). In the DRGC group, the abundance of Fusobacteriota and Euryarchaeota decreased, whereas Firmicutes, Bacteroidota, Halobacterota, Actinobacteriota, and Chloroflexi increased compared to the GC group (Table S2). The gut of grass carp contained nine predominant bacterial families, including Fusobacteriaceae, Halomicrobiaceae, Brevinemataceae, Aeromonadaceae, and Lachnospiraceae (Figure 3B). Compared with the GC group, Halomicrobiaceae and Competibacteraceae showed increased abundance (Table S3). The functional prediction of the bacterial structure indicated that the DRGC group’s intestinal tract had a higher relative abundance of Gram-negative bacteria and a lower relative abundance of Gram-positive, facultatively anaerobic, and anaerobic bacteria compared to the GC group (Figure 4A–D). Moreover, microbiome phenotype predictions indicated that changes in bacterial diversity may primarily influence immune regulation (Figure 4E).

### 2.4. Metabolome Analysis

**Figure 5:**
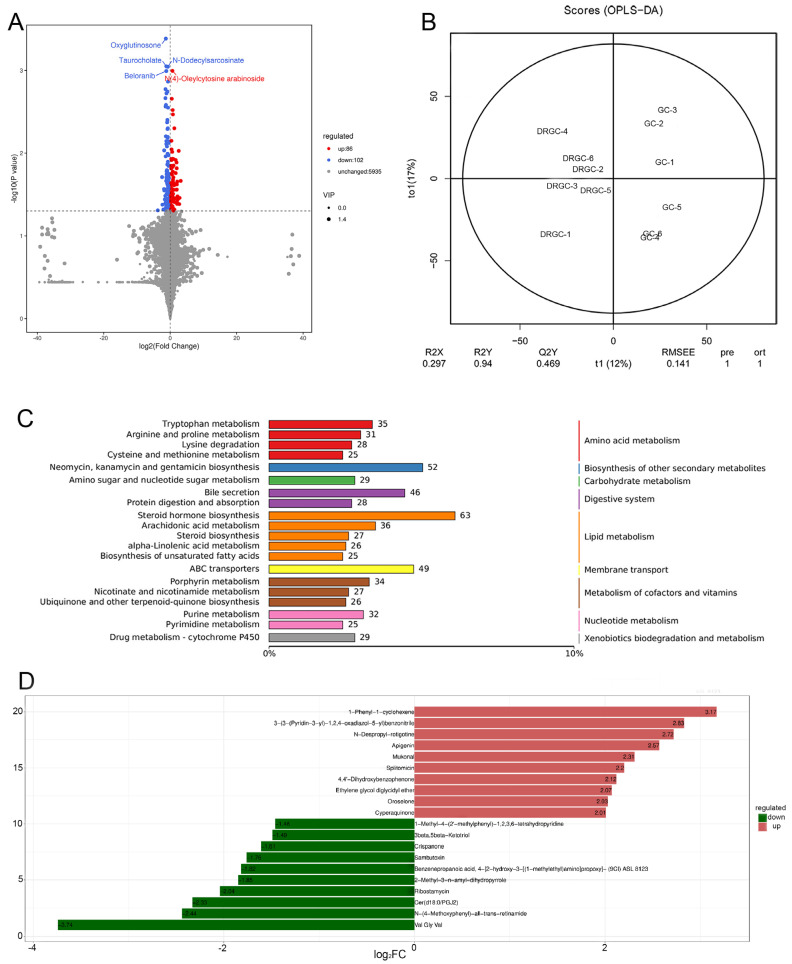
Metabolites between DRGC and GC groups. (**A**) Volcanic map (n = 6 fish per group); (**B**) orthogonal partial least squares−discriminant analysis (OPLS−DA); (**C**) Kyoto Encyclopedia of Genes and Genomes (KEGG) analysis; and (**D**) VIP bar graph. The top 20 differential metabolites were identified between the disease−resistant grass carp (DRGC) and grass carp (GC) groups.

Twelve fecal samples from the DRGC and GC groups were subjected to non-targeted metabolomics analysis using liquid chromatography–mass spectrometry (LC-MS). According to the mass spectrometry analysis, 3628 metabolites were identified in the positive ion mode (Table S4), and 2495 metabolites were found in the negative ion mode (Table S5). Orthogonal partial least squares–discriminant analysis (OPLS-DA) highlighted a clear differentiation between the two groups (Figure S1 and Figure 2). In comparison to the GC group, the DRGC group exhibited a significantly higher count of downregulated metabolites compared to upregulated ones (Figure 5A). The partial least squares–discriminant analysis (PLS-DA) score chart visually demonstrates the model’s classification effectiveness. The DRGC and GC group samples were distinctly separated, suggesting a significant classification effect (Figure 5B). The Kyoto Encyclopedia of Genes and Genomes (KEGG) pathway analysis revealed notable enrichment in lipid, nucleotide, and amino acid metabolism (Figure 5C). The study analyzed differential metabolites between the DRGC and GC groups, exploring the contribution of these metabolites to the observed differences. The primary differential metabolites between the two groups were Val Gly Val, 1-Phenyl-1-cyclohexene, 3-(3-(Pyridin-3-yl)-1,2,4-oxadiazol-5-yl) benzonitrile, N-Despropyl-rotigotine, and Apigenin (Figure 5D).

### 2.5. Transcriptome

Six cDNA libraries were constructed and sequenced. Table 2 displays a summary of the characteristics of these libraries. Post-quality control, each library contained between 43,187,696 and 50,043,924 clean reads, with Q30 values exceeding 96.15%. Subsequently, the clean reads were mapped to the grass carp reference genome, with mapping rates ranging from 91.04% to 92.5% across different libraries.

### 2.6. Identification of Differentially Expressed Genes (DEGs)

**Figure 6:**
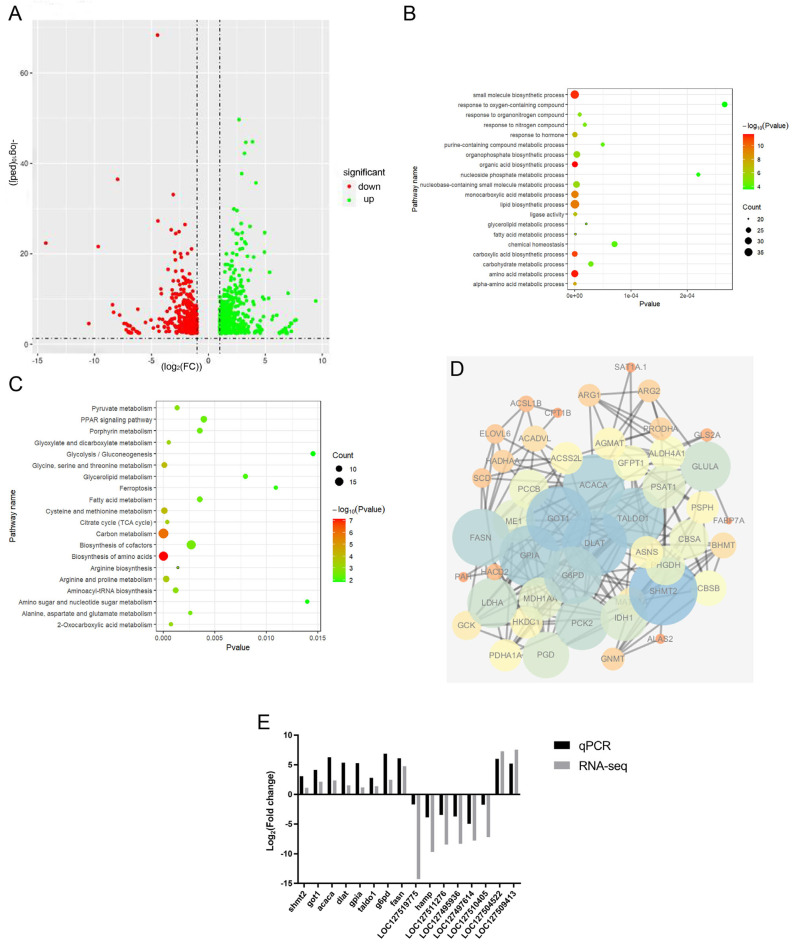
Summary of differentially expressed genes (DEGs) in disease-resistant grass carp. (**A**) Volcano plots of DEGs between disease−resistant grass carp (DRCG) and grass carp (GC) groups. Green and red dots indicate upregulated and downregulated genes, respectively. (**B**) Gene Ontology (GO) annotation of differentially expressed genes (DEGs). (**C**) Kyoto Encyclopedia of Genes and Genomes (KEGG) analysis. (**D**) PPI network and enrichment analysis of hub genes. (**E**) RT−qPCR validation of DEGs. *SHMT2*: serine hydroxymethyltransferase 2; *GOT1*: glutamic−oxaloacetic transaminase 1; *ACACA*: acetyl−CoA carboxylase alpha; *DLAT*: dihydrolipoamide S−acetyltransferase; *GPIA*: glucose-6-phosphate isomerase a; *TALDO1*: transaldolase 1; *G6PD*: glucose−6−phosphate dehydrogenase; *FASN*: fatty acid synthase.

DEGs between the GC and DRGC groups were identified by DESeq2 differential expression analysis and visualized with volcano plots (Figure 6A). A total of 954 DEGs were identified, including 396 that were upregulated and 558 that were downregulated. Thereafter, Gene Ontology (GO) and KEGG pathway enrichment analyses were conducted to identify the biological functions of DEGs between the GC and DRGC groups. The predominant GO terms included lipid biosynthetic process, small-molecule biosynthetic process, and monocarboxylic acid metabolic process (Figure 6B). Based on the KEGG pathway classification, twenty-five pathways were associated with metabolism; in contrast, one pathway each was linked to genetic information processing, cellular processes, and organismal systems. KEGG enrichment analysis revealed that DEGs were predominantly associated with pathways such as carbon metabolism, amino acid biosynthesis, glycine, serine, and threonine metabolism, cysteine and methionine metabolism, arginine and proline metabolism, the citrate cycle (TCA cycle), and glyoxylate and dicarboxylate metabolism (Figure 6C). Hub genes linked to DRGC immunity were identified, and a PPI network of immune-related genes detected in the GO and KEGG analyses and the previous literature was constructed using the STRING tool and analyzed with Cytoscape software (v3.7.1). Following the PPI network analysis, eight genes (*SHMT2* (serine hydroxymethyltransferase 2), *GOT1* (glutamic-oxaloacetic transaminase 1), *ACACA* (acetyl-CoA carboxylase alpha), *DLAT* (dihydrolipoamide S-acetyltransferase), *GPIA* (glucose-6-phosphate isomerase a), *TALDO1* (transaldolase 1), *G6PD* (glucose-6-phosphate dehydrogenase), and *FASN* (fatty acid synthase)) were identified as hub genes due to their interaction degrees exceeding 16 (Figure 6D, Table S6).

### 2.7. Verification

To ensure the accuracy of the RNA-seq results, the expression of 16 DEGs was measured by qRT-PCR using the same RNA samples that were used for the sequencing database. The primer sequences of the identified DEGs are listed in Table S7. The analysis of the 16 DEGs using qRT-PCR showed similar results to the RNA-seq analysis (Figure 6E).

### 2.8. Multi-Omic Joint Analysis

#### 2.8.1. Correlation Analysis Between Microbiome and Metabolome

**Figure 7:**
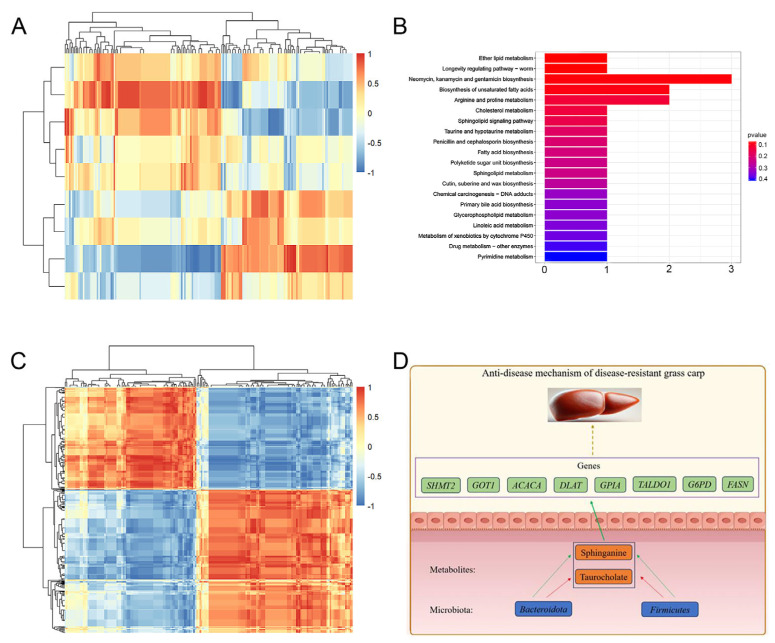
Correlation analysis amongst the microbiome, metabolome, and transcriptome. (**A**) Spearman’s correlation of phyla−level gut microbiota with differential metabolites; (**B**) Kyoto Encyclopedia of Genes and Genomes (KEGG) pathway enrichment analysis of metabolites; (**C**) Spearman’s correlation of metabolites with differential genes; and (**D**) anti−disease mechanism of disease-resistant grass carp. The green arrows represent positive correlations, and the red arrows represent negative correlations.

Differences were observed in the gut microbiota and metabolic characteristics between GC and DRGC. A correlation analysis was performed to investigate the relationship between potential metabolites and key gut microbiota at the phylum level. Figure 7A illustrates a heatmap showing Spearman’s correlation analysis between gut microbiota at the phylum level and metabolites (Table S8). The study revealed a significant association between 9 gut microbiota phyla and 188 metabolites. As shown in Figure 7B, 18 metabolites associated with immune-related pathways were identified, including those involved in ether lipid metabolism, neomycin, kanamycin, and gentamicin biosynthesis, unsaturated fatty acid biosynthesis, arginine and proline metabolism, cholesterol metabolism, sphingolipid signaling, taurine and hypotaurine metabolism, penicillin and cephalosporin biosynthesis, fatty acid biosynthesis, sphingolipid metabolism, primary bile acid biosynthesis, glycerophospholipid metabolism, linoleic acid metabolism, and pyrimidine metabolism. Taurocholate showed a negative correlation with Firmicutes and a positive correlation with Bacteroidota among the metabolites in these pathways. Sphinganine demonstrated a positive correlation with both Firmicutes and Bacteroidota.

#### 2.8.2. Correlation Analysis Between Metabolome and Transcriptome

To explore the relationship between metabolites and genes, a correlation analysis was performed on the metabolome and transcriptome. In addition, a correlation analysis between metabolites and differentially expressed genes was carried out using Spearman’s correlation coefficient (Figure 7C) (Table S9). Taurocholate and sphinganine showed positive associations with the eight immune-related genes (*SHMT2*, *GOT1*, *ACACA*, *DLAT*, *GPIA*, *TALDO1*, *G6PD*, and *FASN*). These genes can be enriched in the immune-related pathway of arginine and proline metabolism.

## 3. Discussion

Fish intestinal traits are influenced not only by feeding habits but also by host genetics [27,28]. In our study, we observed significant differences in intestinal morphology (e.g., villus length and gut wall thickness) between DRGC and GC, which aligns with the findings of Li et al. [29] on gut structural alterations in hybrid fish progenies. We further demonstrate that these hybridization-induced morphological changes may drive shifts in gut microbiota composition [30], as evidenced by distinct microbial community structures between DRGC and GC. Our results corroborate the findings of previous studies [31,32] showing host genetics as a key determinant of microbial diversity.

The gut microbiota plays a pivotal role in immune homeostasis [33,34]. In the present study, we identified Firmicutes and Bacteroidetes as dominant phyla in DRGC, consistent with reports in healthy aquaculture species [35,36,37]. Notably, we observed a significant increase in Firmicutes (including probiotic genera Bacillus and Lactobacillus) and Bacteroidetes in DRGC compared to GC. This finding is particularly relevant because Bacillus and Lactobacillus are well-documented enhancers of disease resistance and immune function in fish [38,39,40,41]. For example, dietary supplementation with *Bacillus amyloliquefaciens* improved immune responses in yellow catfish [42], while Lactobacillus strains boosted immunity in *Nile tilapia* and *Penaeus vannamei* [43,44]. Our data extend these observations by showing that the DRGC’s enriched Firmicutes/Bacteroidetes may synergistically enhance immune capacity through microbial–host interactions.

Through integrated metabolomic and transcriptomic analyses, we identified taurocholate and sphinganine as key immune-modulating metabolites in DRGC. Specifically, taurocholate exhibited anti-inflammatory properties akin to its role in murine colitis models [45], while sphinganine influenced immune cell dynamics via sphingolipid synthesis [46,47]. Importantly, we discovered that these metabolites were strongly correlated (Spearman’s analysis) with eight immune-related genes in DRGC: *SHMT2*, *GOT1*, *ACACA*, *DLAT*, *GPIA*, *TALDO1*, *G6PD*, and *FASN* (Figure 7D). *SHMT2*, a key enzyme in one-carbon metabolism, may be associated with the immunotherapy response in patients with renal cancer [48]. Additionally, *GOT1* encodes cytosolic aspartate aminotransferase, a key MAS enzyme facilitating the reversible amino group transfer between glutamate and aspartate [49]. Xu et al. [50] demonstrated that the metabolic enzyme GOT1 is crucial for the proliferation and effector functions of effector CD^8+^ T cells, particularly under serine-free conditions, due to its upregulation. In triploid crucian carp, *GOT1* potentially regulates innate immunity by influencing the biosynthesis and transformation of key antibiotics and antimicrobial peptides [51]. *ACACA* is typically found in lipogenic tissues such as liver and adipose [52] and is associated with immune response [53]. *DLAT* expression showed a positive correlation with various immunological features, including immune cell infiltration, the cancer-immunity cycle, pathways predicted for immunotherapy, and inhibitory immune checkpoints [54]. Furthermore, Chen et al. [55] reported that the *DLAT* and *LDHA* genes potentially modulate the immune microenvironment in dilated cardiomyopathy by affecting activated dendritic cells, activated mast cells, and M0 macrophages. Glycoprotein Ia (*GPIA*), or integrin alpha 2 (*ITGA2*) [56], facilitates cell adhesion to the extracellular matrix and is crucial for bidirectional signaling, cell motility, stemness, and angiogenesis [57]. Variations in *ITGA2* expression subtly and dynamically influence the tumor immune microenvironment and immunogenicity [58]. *ITGA2* is reported to significantly influence the innate immune response in fish [59]. *TALDO1*, an essential enzyme in the pentose phosphate pathway, facilitates the production of ribo-5-phosphate (R5P) for nucleic acid synthesis and nicotinamide adenine dinucleotide phosphate (NADPH) for lipid biosynthesis [60]. Cen and Lu [61] performed functional enrichment and immune infiltration analysis and revealed that TALDO1 negatively regulates the immune response. The liver’s production of plasma proteins, such as acute phase reactants, cytokines, and complement factors, highlights the importance of *G6PD* function in systemic immunity [62]. *G6PD*-deficient hepatocytes show altered transcriptomic networks in redox and immune response pathways when exposed to oxidant stress [63]. *FASN* is a crucial enzyme for the de novo synthesis of long-chain fatty acids [64]. *FASN* regulates immune cell survival, activation, differentiation, and function. Consequently, *FASN* contributes to the onset and progression of various conditions, including tumors, cardiovascular diseases, inflammatory diseases, autoimmune diseases, infectious diseases, and other pathological states [65]. Comparative analysis revealed significant upregulation of the eight immune-associated genes in DRGC compared to GC, suggesting their collective contribution to enhanced immunocompetence against pathogenic challenges. This integrated gene–metabolite network provides mechanistic insights into DRGC’s superior disease resistance, potentially mediated through synergistic regulation of immune–metabolic pathways, antimicrobial peptide synthesis, and cellular immunity optimization.

Our study highlights the gut–liver axis as a critical mediator of DRGC immunity. The observed correlations between gut microbiota (Firmicutes/Bacteroidetes), liver metabolites (taurocholate/sphinganine), and liver immune genes suggest bidirectional crosstalk. While previous studies focused on toxin-induced gut–liver axis disruption [66,67,68,69,70,71], our work uniquely demonstrates its physiological role in hybrid fish immunity. Specifically, we propose that DRGC’s enriched probiotics (Firmicutes and Bacteroidetes) may prime hepatic immune gene expression via metabolite signaling and that taurocholate/sphinganine act as molecular bridges between intestinal microbes and liver immunity (Figure 7D). This model diverges from toxin-centric studies by emphasizing immune optimization through host–microbe coevolution.

In conclusion, our multi-omic investigation demonstrates that hybridization drives functional optimization of the gut–liver axis in DRGC through three interconnected mechanisms. Firstly, morphological adaptations in intestinal architecture facilitate the colonization of beneficial microbial communities. Secondly, metabolic reprogramming mediated by taurocholate and sphinganine reinforces immune modulation. Thirdly, transcriptional activation of key immune-related genes (*SHMT2*, *FASN*, etc.) enhances pathogen resistance (Figure 7D). Collectively, these findings establish a novel framework for understanding hybrid vigor in aquaculture species, wherein genetic hybridization orchestrates gut–liver axis remodeling to achieve superior disease resilience.

## 4. Materials and Methods

### 4.1. Animal Materials

Grass carp and disease-resistant grass carp (300 ± 20 g) were sourced from the Engineering Research Center of Polyploid Fish Reproduction and Breeding of the State Education, Ministry, College of Life Sciences, Hunan Normal University, China. Prior to the experiment, the fish were acclimated for one week in a pathogen-free laboratory environment maintained at 22 ± 1 °C. Fecal samples from 6 grass carp and 6 disease-resistant grass carp were collected in sterile centrifuge tubes, rapidly frozen in liquid nitrogen, and stored at −80 °C for subsequent DNA extraction and microbiome/metabolomic analysis. Meanwhile, livers were collected from three randomly selected specimens in each grass carp group. The samples were promptly frozen using liquid nitrogen and stored at −80 °C for subsequent transcriptomic analysis.

### 4.2. Determination of the Morphological Structure of Intestinal Tissue

Six grass carp and six disease-resistant grass carp were chosen, and their midguts were removed and placed in 4% tissue fixative (P1110, Solarbio Corp., Beijing, China). The tissues were dehydrated, wax-embedded, sectioned, and stained with hematoxylin and eosin (H&E). NDP.view2 software facilitated the measurement and analysis of intestinal villus height, villus width, and intestinal wall thickness across the two groups.

### 4.3. 16S rRNA Gene Sequencing Analysis

Total genomic DNA was extracted from the samples following the manufacturer’s protocol of the TGuide S96 Magnetic Soil/Stool DNA Kit (Tiangen Biotech, Beijing, China). Subsequent 16S rRNA sequencing and bioinformatics analyses were conducted based on standardized methodologies adapted from previously published approaches [72]. Key experimental steps included the following:

The sequencing library was constructed by Biomarker Technologies Corporation (Beijing, China) for paired-end sequencing on the Illumina NovaSeq 6000 platform. Amplification of the V3-V4 hypervariable region of the 16S rRNA gene was achieved using universal primers 338F (5′-ACTCCTACGGGAGGCAGCAG) and 806R (5′-GGACTACHVGGGTWTCTAAT). Raw sequencing data underwent quality control processing through Trimmomatic (v0.33), followed by primer trimming with Cutadapt (v1.9.1). Paired-end reads were merged and subjected to chimera removal via UCHIME (v8.1) to generate high-quality clean reads. These sequences were clustered into operational taxonomic units (OTUs) at a 97% similarity threshold using the USEARCH pipeline (v10). Taxonomic classification of representative OTU sequences was performed in QIIME2 (v2020.6) through a naïve Bayesian algorithm against the SILVA database (Release 138). Beta diversity analysis was carried out via principal coordinate analysis (PCoA) to evaluate intergroup differences in microbial community composition. Alpha diversity indices, including ACE and Shannon metrics, were computed using QIIME2. Taxonomic abundance profiles at the order level were statistically analyzed and visualized as heatmaps through R software (v3.5.3). Functional annotation of OTUs was predicted by aligning KEGG pathway databases through PICRUSt: http://picrust.github.io (accessed on 23 October 2024).

### 4.4. Metabolomic Analysis

Untargeted metabolomic analysis of fish fecal samples was performed according to published methods [73], with specific procedures executed as follows: Fecal specimens (50 mg) were homogenized in 400 μL of ice-cold methanol/water (1:1, *v*/*v*) using a vortex shaker (QL-901, Qilin Bell Instruments, China) for 30 s, followed by sequential low-temperature ultrasonication (5 °C, 40 kHz, 30 min), static incubation at −20 °C (30 min), and centrifugation (13,000× *g*, 4 °C, 15 min) in a refrigerated centrifuge (5430R, Eppendorf, Germany). The collected supernatant was nitrogen-evaporated to dryness and reconstituted in 120 μL of acetonitrile/water (1:1, *v*/*v*), with subsequent ultrasonic centrifugation (5 min) prior to LC-MS analysis. Chromatographic separation utilized a Thermo UHPLC system equipped with an ACQUITY BEH C18 column (2.1 mm × 100 mm, 1.7 μm; Waters, USA), employing 0.1% formic acid (aqueous) and acetonitrile/isopropanol (1:1, *v*/*v*, 0.1% formic acid) as mobile phases under the following conditions: 2 μL injection volume, 0.4 mL/min flow rate, and 40 °C column temperature. Mass spectrometric detection was conducted via a Thermo UHPLC-Q instrument with electrospray ionization, configured with ion source parameters (400 °C temperature; sheath/auxiliary gas flows: 40/30 psi), voltage settings (−2800 V negative mode; 3500 V positive mode), and normalized collision energy gradients (20–40–60 V). Raw data processing through the BMK Cloud platform identified differential metabolites (VIP > 1, *p* < 0.05) using Student’s *t*-test and multivariate ANOVA statistical approaches.

### 4.5. Transcriptome Analysis

Total RNA extraction from liver tissues was conducted with TRIzol reagent (Invitrogen), followed by RNA integrity assessment (RIN > 8.0) and quantification using an Agilent 2100 Bioanalyzer (Santa Clara, CA, USA) coupled with a NanoDrop 2000 spectrophotometer (Thermo, MA, USA). Six RNA-seq libraries representing two experimental groups underwent polyadenylated mRNA enrichment, fragmentation, and double-stranded cDNA synthesis prior to paired-end sequencing (2 × 150 bp) on an Illumina NovaSeq 6000 platform. Raw sequencing reads were processed with Fastp (v0.23.4) for adapter trimming and quality filtering, followed by integrity validation through FastQC (v0.12.1). HISAT2 (v2.2.0) aligned clean reads to the *Ctenopharyngodon idella* reference genome (GenBank accession: GCF_019924925.1), with gene expression quantified as FPKM (fragments per kilobase million). Differential expression analysis between DRGC and GC groups was implemented via DEGSeq2 (v1.36) under thresholds of |log2(fold change)| > 1 and false discovery rate (FDR) < 0.05. Functional enrichment analyses (Gene Ontology and KEGG pathways) were performed with ClusterProfiler (v3.6.0), retaining terms with *p* < 0.05 after Benjamini–Hochberg correction. Immune-related DEGs were identified through integrated annotation from GO/KEGG analyses and literature mining. Protein–protein interaction networks were reconstructed using STRING (v11.5), with topological analysis in Cytoscape (v3.9.1) determining hub genes based on maximal connectivity degrees.

### 4.6. Verification of Transcriptome Data

To validate transcriptomic sequencing data from DRGC vs. GC comparisons, qRT-PCR was employed to assess 16 DEGs. First-strand cDNA synthesis was performed with the PrimeScriptTM RT reagent kit (Takara, China) following the manufacturer’s guidelines. Target-specific primers for selected DEGs and the reference gene *β-actin* (Table S7) were utilized to establish a 10 μL reaction system containing 5 μL SYBR Green qPCR Master Mix, 0.5 μL each of forward/reverse primers (20 μM), 1 μL diluted cDNA (1:5), and 3 μL nuclease-free water. Amplification protocols comprised an initial denaturation at 95 °C (2 min), followed by 40 cycles of 95 °C (15 s) and 60 °C (30 s) for annealing/extension. Each biological sample was analyzed in triplicate technical replicates. The relative quantification of mRNA expression levels was determined through the 2^−ΔΔCt^ method, with *β-actin* serving as the endogenous normalization control.

### 4.7. Statistical Analysis

All quantitative data were statistically processed using IBM SPSS Statistics (v26.0, Armonk, NY, USA) under rigorous analytical protocols. Intergroup comparisons were evaluated through two-tailed Student’s *t*-test with Bonferroni correction, with significance defined at the α = 0.05 threshold. Continuous variables are presented as arithmetic mean ± standard deviation from six independent biological replicates. Bivariate correlations were quantified via Spearman’s rank-order coefficient, incorporating non-parametric adjustments for tied ranks.

## Figures and Tables

**Table 1:** Intestinal histological results of disease-resistant grass carp (DRGC) and grass carp (GC) groups.

Parameters	DRGC	GC
Villus length (μm)	533.35 ± 22.43 ^a^	376.81 ± 21.23
Thickness of gut wall (μm)	77.36 ± 4.22 ^a^	57.81 ± 4.77
Villus width (μm)	79.60 ± 6.53 ^a^	109.45 ± 9.30

Values are presented as mean ± SD (n = 6). ^a^ indicates significant difference (*p* < 0.05).

**Table 2:** Summary statistics of transcriptome sequences.

Sample	Clean Reads	GC Content (%)	Q30 (%)	Uniquely Mapped Reads (Ratio)	Multiple Mapped Reads (Ratio)	Total Mapped Reads (Ratio)
DRGC-1	50,043,924 (99.80%)	46.52	96.16	43,520,258 (86.96%)	2,771,962 (5.54%)	46,292,220 (92.5%)
DRGC-2	47,104,516 (99.79%)	46.41	96.20	40,953,442 (86.94%)	2,490,242 (5.29%)	43,443,684 (92.23%)
DRGC-3	45,800,350 (99.81%)	46.56	96.25	39,678,432 (86.63%)	2,449,190 (5.35%)	42,127,622 (91.98%)
GC-1	44,884,616 (99.82%)	46.57	96.19	38,596,774 (85.99%)	2,267,454 (5.05%)	40,864,228 (91.04%)
GC-2	47,683,850 (99.80%)	46.70	96.22	40,773,060 (85.51%)	2,640,728 (5.54%)	43,413,788 (91.05%)
GC-3	43,187,696 (99.85%)	46.62	96.56	37,276,028 (86.31%)	2,461,866 (5.70%)	39,737,894 (92.01%)

DRGC, disease-resistant grass carp; GC, grass carp.

## Data Availability

The original data presented in the study are openly available in the China National Center for Bioinformation (CNCB) database under accession number PRJCA034676.

## Supplementary Materials

The supplementary information was uploaded to FigShare (https://doi.org/10.6084/m9.figshare.28216601; https://doi.org/10.6084/m9.figshare.28216748).
